# Comparative analysis and directed protein evolution yield an improved degron technology with minimal basal degradation, rapid inducible depletion, and faster recovery of target proteins

**DOI:** 10.21203/rs.3.rs-5348956/v1

**Published:** 2024-11-15

**Authors:** Mazhar Adli, De Xing, Tao Bai, Ozlem Neyisci, Seyedehzahra Paylakhi, Alexander Duval, Yasemin Tekin

**Affiliations:** Northwestern University, Feinberg School of Medicine; Northwestern University, Feinberg School of Medicine; Northwestern University, Feinberg School of Medicine; Northwestern University, Feinberg School of Medicine; Northwestern University, Feinberg School of Medicine; Northwestern University, Feinberg School of Medicine; Northwestern University, Feinberg School of Medicine

## Abstract

Biological mechanisms are inherently dynamic, requiring precise and rapid gene manipulation for effective characterization. Traditional genetic perturbation tools such as siRNA and CRISPR knockout operate on timescales that render them unsuitable for exploring dynamic processes or studying essential genes, where chronic depletion can lead to cell death. Here, we compared four major inducible degron systems—dTAG, HaloPROTAC, and two auxin-inducible degron (AID) tools—in human pluripotent stem cells. We evaluated basal degradation levels, inducible degradation kinetics, and recovery dynamics for endogenously tagged genes. While the AID 2.0 system is the most efficient for rapid protein degradation, it exhibited higher basal degradation and slower recovery after ligand washout. To address these challenges, we applied directed protein evolution, incorporating base-editing-mediated mutagenesis and iterative functional selection and screening. We discovered novel OsTIR1 variants, including S210A, with significantly enhanced overall degron efficiency. The resulting system, designated as AID 3.0, demonstrates minimal basal degradation and rapid and effective target protein depletion and substantially rescues the cellular and molecular phenotypes due to basal degradation or slow target protein recovery in previous systems. We conclude that AID 3.0 represents a superior degron technology, offering a valuable tool for studying gene functions in dynamic biological contexts and exploring therapeutic applications. Additionally, the research strategy used here could be broadly applicable for improving other degron and biological tools.

## Introduction

Technological advancements in molecular tools that enable gene function loss are essential for understanding biological mechanisms. To fully comprehend a biological system, we must manipulate its essential components and assess the phenotypic changes resulting from these perturbations. Methods like siRNA knockdown and CRISPR-based gene knockout are critical for targeted and large-scale genetic manipulations. However, these approaches have notable limitations when studying gene functions in detail. Firstly, they are unsuitable for examining highly dynamic biological processes regulated by a gene, as achieving a near-complete null allele phenotype requires considerable time. siRNA or shRNA-based gene knockdowns usually take 4–5 days, while CRISPR knockouts often require more than a week for significant gene editing and protein depletion at the population level. Generating a clonal population can extend this timeframe to several months. Secondly, these genetic perturbations are not universally applicable to all protein-coding genes. For example, they cannot be used to study essential human genes whose chronic depletion causes cellular lethality^1^. Recent CRISPR-based large-scale perturbation studies, such as the Cancer Dependency Map, have identified around 2000 genes essential for cellular viability across various human cell lines^2,3^. Thirdly, and most crucially, the chronic and extended nature of these genetic perturbations can induce compensatory genetic mechanisms^4,5^. This well-documented but frequently overlooked issue can significantly hinder the interpretation of null allele (i.e., gene knockout or knockdown) phenotypes and obscure our understanding of the molecular mechanisms regulated by specific genes.

An ideal genetic manipulation approach should possess four characteristics: 1. It should be rapidly inducible, thereby minimizing genetic compensation. 2. It should be tunable, allowing control over the level of target protein depletion to study phenotypes across various protein dosages. 3. The null allele phenotype should be rapidly reversible, enabling rescue experiments within the same biological system, such as a clonal cell line. 4. The approach should be universally applicable to all genes. In theory, ligand-inducible targeted protein degradation methods meet nearly all of these criteria. As a result, these methods have become indispensable tools in basic scientific research and hold tremendous potential for therapeutic applications^6–8^.

Excitingly, several chemically inducible targeted protein degradation approaches have been developed. These technologies require a specific “degron”—a sequence of amino acids added to the target protein—that can be recognized and degraded by the E3 ligase-mediated ubiquitin-dependent proteasomal degradation system (UPS). Notably, although some of these methods were established over 16 years ago^8,9^, advancements in CRISPR-based genome editing have significantly expanded their utility. This is because the degron sequence can now be inserted into endogenous genes’ N- or C-terminal regions via CRISPR-mediated homology-directed repair ^10,11^.

Once tagged with the degron, the degradation of endogenous proteins can be induced through the UPS with the aid of a “ligand” molecule, which acts as a bridge between the protein of interest and the endogenous E3 ligases and ubiquitin degradation system. Among the targeted protein degradation strategies, the auxin-induced degron (AID) systems, which use exogenous plant-derived TIR1^9,10,12,13^ or AFB2^14^ adapter proteins, and the degradation tag dTAG^15^ and HaloPROTAC^16,17^systems, which rely on endogenous E3 ubiquitin ligase components, are the most widely used strategies.

The advantages and limitations of these tools are yet to be clearly documented. Previous comparison has been limited to specific tools such as AID 2.0 vs ATFB2^12^. More recently, a more systematic approach was used to compare four different degron technologies^18^. However, in this study, a previous version of the AID tool was used, and all comparisons were made using exogenously overexpressed degron-tagged cDNA rather than endogenous genes. Here, we intially established and comparatively assessed the newest versions of all four major degron technologies in human pluripotent cells using endogenous genes. We used a directed protein evolution approach to re-engineer the relatively more efficient degron system to overcome the basal degradation and target recovery issues after ligand washout. To compare all the tools, we used CRISPR to homozygous knock-in the needed degrons for all tools at the end of the same genes. We then assessed their relative efficiencies in basal (i.e., uninduced) degradation, the kinetics of inducible degradation, and the dynamics of reversibility and target protein recovery after ligand washout. This comprehensive analysis highlights critical differences between the methods and their respective advantages. These comparative analyses nominated the auxin-inducible OsTIR1 system as the most efficient tool, with faster kinetics for inducible complete degradation. However, the high kinetic efficiency also brought limitations, such as higher basal degradation and slower recovery rates. To address these issues, we engineered the AID 2.0 system through directed protein evolution. This process involved saturating mutagenesis of the OsTIR1 protein using a custom-designed sgRNA library with Cytosine^19,20^ and Adenine^21^ base editors (BE). This in vivo hypermutation and directed evolution strategy resulted in several mutations that address the critical limitations of the original OsTIR1 system. Therefore, we present the AID 3.0 technology, demonstrating minimal basal degradation, efficient target protein depletion, and faster dynamics of target protein recovery after ligand washout.

## Results

### Comparative analysis of four different inducible degron technologies:

**Figure 1:**
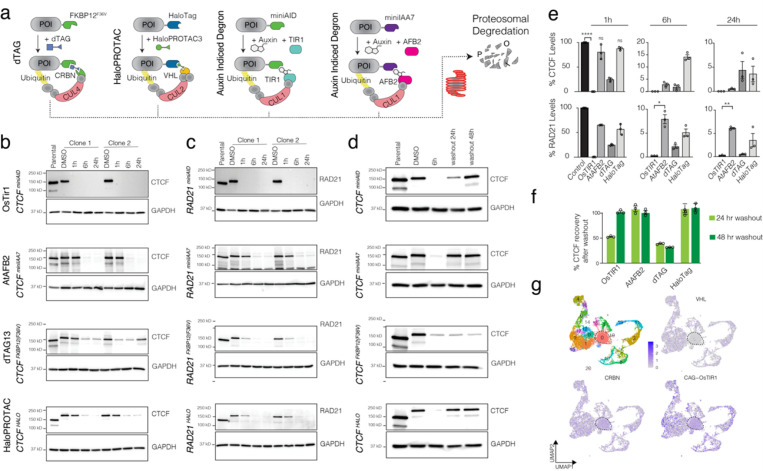
Comparative analysis of major targeted protein depletion strategies. **a)** Schematic illustrates the four degron technologies for ligand inducible protein depletion. **b-c)** Western blots indicate comparative depletion of endogenously tagged CTCF and RAD21 proteins in KOLF2.2J iPSC. The endogenous genes were homozygously knocked in with miniAID, miniIAA7, FKBP12(F36V) or HaloTag degron tags and the target proteins were induced to degrade by treating cells with 1 mM 5-Phenyl-indole-3-acetic acid (5-Ph-IAA, synthetic auxin), 500 mM Indole-3-acetic acid (IAA, auxin), 1 mM dTAG13 or 1 mM HaloPROTAC3, respectively, for 0 hr, 1 hr, 6 hr and 24 hr using western blots. **d)** Western blots show relative recovery rates of endogenous CTCF protein levels in four degron-based technologies. KOLF2.2J iPSC were treated with respective ligands for 6 hr, then the ligands were washed and target protein levels were assessed after 24 hrs and 48 hrs after the ligand washout. **e)**Bar plots show quantification of target protein depletion after 1 hr, 6 hr and 24 hrs of treatment with each chemical degrader. **f)**The bar plot shows relative CTCF protein recovery after ligand washout I each degron system. Data is represented as mean values ± SD (*n*=3). NS represents not significant, * represents *P* < 0.05, ** represents *P* < 0.01, *** represents *P* < 0.001, **** represents *P* < 0.0001, student *t*-test. **g)**The single cell analyses (UMAP visualization) shows the relative expression levels of endogenous *VHL*, *CRBN*genes and CAG-promoter driven *OsTIR1*gene expression in iPS cells differentiated as embryoid bodies for 21 days.

Initially, we set up a robust system to assess the relative efficiency of each tool. To minimize cell line bias in our study, we established all four degron systems (Fig. 1a) in the same open-access KOLF2.2J iPSC line. The dTAG approach^15^ utilizes the synthetic ligand AP1867 to deplete FKBP12^F36V^-degron-tagged target proteins via the Cereblon (CRBN) E3 ubiquitin ligase complex. In contrast, the HaloPROTAC system^17^ uses a bifunctional ligand to target HaloTag7-fusion proteins for degradation through the VHL E3 ubiquitin ligase complex.

Unlike these systems, which rely on endogenous E3 ligases, the auxin-inducible degron (AID) systems require the exogenous expression of E3 ligase adapter proteins, such as TIR1 from *Oryza sativa* (OsTIR1) or AFB2 from *Arabidopsis thaliana* (AtAFB2). The original OsTIR1 system had significant leaky degradation and required high auxin concentrations. However, a single point mutation in OsTIR1 (F74G or F74A) has substantially addressed these issues^12,13^. Consequently, we knocked in OsTIR^F74G^ and AtAFB2^F74G^ into the AAVS1 safe harbor locus to ensure higher expression levels driven by the synthetic CAG promoter^22^.

We focused on RAD21 and CTCF, critical transcriptional regulators that have been extensively studied using various degron technologies to investigate their roles in 3D genome organization^23,24^. Using CRISPR (Cas9/sgRNA RNP combined with degron templates), we introduced the latest degrons into the C-terminal regions of RAD21 and CTCF (Supplementary Fig. 1). We generated several clonal cell lines with homozygous tags for each gene, confirmed by PCR genotyping and Western blot analysis. First, we evaluated the effects of degron tagging on the basal and ligand-induced stability of CTCF (Fig. 1b) and RAD21 (Fig. 1c). Each inducible system is utilizing a degron of different size and different ligands (Supplementary Fig. 2). After inducing with indicated ligand concentrations, we measured endogenous protein levels at 1, 6, and 24 hours post-ligand induction. Additionally, we assessed the impact of ligands on iPSC cell pluripotency, viability, and proliferation, finding no significant effects at the tested auxin doses (5-Ph-IAA at 1 μM and IAA at 500 μM). Notably, at the suggested doses of 1 μM, we observe that both dTAG13^15,25^ and HaloPROTAC3^16,17^ substantially reduced the iPSC cell proliferation (Supplementary Fig. 3). A critical feature of any degron technology is its reversibility. We, therefore, measured the recovery of endogenous CTCF protein following a 6-hour ligand treatment, with subsequent assessments of protein levels at 24 and 48 hours after ligand washout (Fig. 1d).

We investigated the kinetics of target protein depletion across multiple clonal cell lines using various degron systems. While all systems achieved significant target protein reduction within 24 hours, notable differences in depletion rates emerged for earlier time points (Fig. 1e). The OsTIR system consistently demonstrated superior depletion efficiency compared to the other methods, whereas HaloTag exhibited substantially slower kinetics. Crucially, despite its rapid depletion kinetics, the OsTIR1 and dTAG systems showed slower target protein recovery after a 24-hour of ligand washout (Fig. 1f). Notably, after 48 hours, all systems fully restored target protein levels, with the exception of dTAG, where the target protein was never recovered. To validate these findings further, we also compared the recovery rates of RAD21, whose long-term depletion is lethal in iPSC cells. Supporting the recovery rates of CTCF, we observed the lowest number of cell survival (after ligand treatment for 6 hrs and washout) in dTAG system (Supplementary Fig. 4), indicating the target protein is not recovered after the ligand washout in this system.

Each degron system relies on distinct E3 ligases, which may display tissue-specific expression patterns^7^. Therefore, the target protein depletion efficiency could be significantly influenced by the levels of these E3 ligases. To explore this hypothesis, we examined the expression of adapter proteins in pluripotent iPSCs and various differentiated states at a single-cell resolution. Differentiating iPSCs into embryoid bodies over 21 days revealed 20 distinct cellular clusters, as determined by single-cell RNA sequencing (scRNA-Seq). We observed near-uniform expression of endogenous CRBN (dTAG) and CAG-promoter-driven OsTIR1 across clusters (Fig. 1g). In contrast, VHL (HaloPROTAC3) E3 ligase expression varied considerably among clusters, with VHL being nearly absent in the largest cluster (Cluster 0). These findings indicate that VHL-dependent target protein depletion might be restricted to specific cell types, highlighting the potential limitations imposed by the detection thresholds and sparsity of scRNA-Seq data.

### Base Editing-Mediated Mutagenesis Screening to Evolve an Improved AID Degron Tool

Our comparative analysis identified the OsTIR1 system as the most efficient degron tool, despite its slower recovery rate. We subsequently employed this system to generate null alleles of essential human genes and study their molecular phenotypes as part of the MorPhic consortium project (Morphic.bio). Notably, we consistently observed efficient and rapid depletion of endogenous target proteins, with some targets being nearly completely depleted in as little as 10 minutes (Supplementary Fig. 5). Critically, we further observed a substantial basal degradation for certain genes. While most degron-tagged endogenous genes exhibited minimal basal degradation, this issue was especially pronounced for certain genes (Supplementary Fig. 5). Basal degradation was a significant limitation of the original AID system. Strategies such as using doxycycline-inducible OsTIR1^10^ and exogenous expression of auxin response transcription factors (ARFs)^26^ have been proposed to address this issue. However, a major advancement was achieved with protein engineering, which led to identifying the F74G/A mutations in OsTIR1, resulting in the development of AID 2.0 that overcame some of the major shortcomings of the original degron^12,13^. This improvement substantially reduced leaky basal degradation and significantly decreased the auxin doses required for efficient target depletion^12,13^. Notably, our results indicate that, despite these substantial advancements, leaky basal degradation persists in AID 2.0, particularly affecting certain proteins.

Our comparative analysis of degron systems and large-scale endogenous protein tagging approaches has established that OsTIR1 AID 2.0 represents a highly effective degron technology. However, despite its high efficiency for on-target degradation, two significant limitations have emerged during our efforts: a protracted recovery period following ligand washout, and notable leaky basal degradation of certain endogenous targets. The absence of superior alternatives led us to develop a directed evolution-based protein engineering strategy to address these shortcomings. To this end, we implemented an unbiased hypermutation strategy utilizing both Cytosine Base Editors (CBE)^20^ and Adenine Base Editors (ABE)^21^, combined with multiple rounds of functional screening to identify mutants with enhanced properties. Base editors, including ABEmax and AncBE4max, facilitate the introduction of single nucleotide changes without causing double-strand DNA breaks or requiring donor DNA templates^20,27^. ABEmax enables A:T to G:C conversions^28^, while AncBE4max promotes G:C to A:T conversions^28^. We constructed a comprehensive sgRNA library targeting OsTIR1 (n = 231 sgRNAs) to achieve extensive mutagenesis with these base editing systems. This library was introduced alongside ABE and CBE into cells expressing OsTIR^F74G^, enabling random incorporation of base edits into *OsTIR1*, which is tandem tagged with GFP and miniAID degron. This approach allowed us to generate a diverse array of amino acid mutations in the OsTIR1 protein, facilitating the identification of variants with improved performance characteristics.

**Figure 2:**
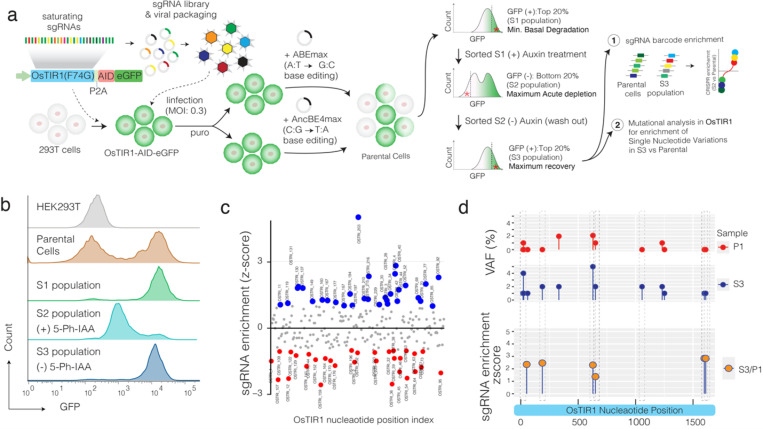
Saturating CRISPR base editing for *in vivo* evolution of adapter protein for efficient auxin-inducible degron technology. **a)** The schematic shows overall directed evolution by saturating CRISPR base editing screening and phenotypic selection strategy of OsTIR(F74G) that will yield a degron system with minimal basal degradation, fast depletion kinetics and faster target protein recovery. **b**) The flow cytometry data shows relative GFP reporter expression after various phenotypic selections. **c)** The enrichment analysis shows relative sgRNA abundance in S3 population compared to the parental cells. **d)** The lollipop plots show variant frequencies of introduced mutations that overlap with the base editing window of the enriched sgRNAs in S3 population compared to the parental cells.

Following the mutagenesis screening, we implemented a multi-step functional screening strategy (Fig. 2a) to identify gain-of-function mutations that met the following criteria: 1) minimal impact on basal levels of the target protein, 2) maintenance of rapid degradation kinetics, and 3) enhanced recovery of the target protein after ligand washout. To achieve this, we utilized flow-assisted cell sorting (FACS) to isolate the top 20% of GFP-positive cells expressing the sgRNA library in conjunction with both base editors. We then subjected these cells to a 1-hour auxin treatment and performed FACS to select the bottom 20% of GFP-negative cells, identifying mutations that still permitted efficient target protein depletion. Following this, we cultured the cells without auxin for 3 days and re-sorted to isolate the top 20% of GFP-positive cells, thus selecting mutations that facilitate faster recovery of the target protein (Fig. 2b).

After completing these three rounds of functional selection and screening, we extracted genomic DNA, PCR-amplified the sgRNA backbone and the OsTIR1 coding sequence, and performed next-generation sequencing (NGS) to identify enriched sgRNAs and *de novo* gain-of-function mutations in OsTIR1. The sgRNA enrichment analysis, using established tools^29^, revealed several sgRNAs that were highly enriched or depleted in the final population compared to the parental cells, indicating the potential success of our screening approach (Fig. 2c). Moreover, variant allele frequency analysis, confirmed through manual inspection of reads in IGV browser, identified several enriched nonsynonymous mutations. To ensure these mutations were introduced by base editors, we overlapped the nonsynonymous mutations with the sgRNA guiding sequence and selected those within the base editing window. This stringent analysis ultimately nominated 10 OsTIR1 mutations as candidates that significantly improve AID technology (Fig. 2d).

#### Generation and Validation of OsTIR1 Mutations

**Figure 3:**
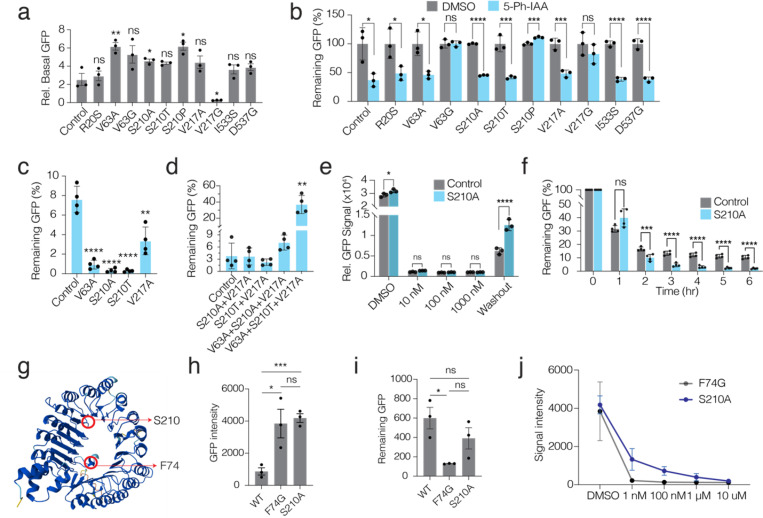
Directed evolution yields novel variants that improve inducible depletion and faster recovery of target proteins. **a)** The bar plots show Incucyte-measured basal GFP levels in HEK239T cells transiently expressing (lipofectamine) degron tagged GFP and ten different point mutations in OsTIR1^F74G^ protein (AID 2.0). **b)** The bar plots show Incucyte-measured GFP levels in HEK293T cells transiently expressing various OsTIR1 variants after treating cells with 1 mM 5-Ph-IAA for 12 h. **c)** The bar plot shows Incucyte-measured degron-tagged GFP reporter depletion in cells expressing the four candidate OsTIR1 point-mutant variants after treatment with 1 mM 5-Ph-IAA for 24 h. **d)** The bar plot shows Incucyte-measured-measured degron-tagged GFP reporter depletion in cells expressing combinatorial mutations in OsTIR1 after treatment with 1 mM 5-Ph-IAA for 24 h. **e)** The bar plot shows Flow-cytometry-measured degron-tagged GFP levels in cells expressing OsTIR1^F74G^ (AID 2.0) and with the newly identified top mutations S210A, OsTIR1^F74G+S210A^ (AID 3.0) after treatment with different 5-Ph-IAA concentrations for 24 hr and after ligand washout. **f)** The bar plot shows Incucyte-measured degron-tagged GFP levels in AID2.0 and AID 3.0 systems after various times of treating with 1 mM 5-Ph-IAA. Plasmids were inserted into AAVS1 safe harbor site in HEK293T cells using CRISPR/Cas9 technology. **g)**, Alphafold2-predicited structure of the OsTIR1. The location of F74 and S210 amino acids are indicated **h-i)** The bar plot shows Flow-cytometry-measured basal (h) or depleted GFP levels after ligand induction (i) in HEK293T cells expressing WT OsTIR1^WT^, OsTIR1^F74G^ or OsTIR^S210A^ (without F74G mutation) mutants. **j)** Line plots show basal and remaining GFP levels of single mutants OsTIR1^F74G^ or OsTIR1^S210A^ after treatment with different auxin concentrations. Data and error bars in (a-f) and (h-i) indicate the means ± SD (*n* = 3–4 independent biological replicates, NS represents not significant, * represents *P* < 0.05, ** represents *P* < 0.01, *** represents *P* < 0.001, **** represents *P* < 0.001, two-tailed *t*-test.

We introduced all ten single amino acid mutations into the OsTIR1^F74G^-T2A-GFP^miniAID^ construct (AID 2.0 system) and confirmed their presence via Sanger sequencing (Supplementary Fig. 7). To functionally validate these mutations, we transiently expressed each variant in HEK293T cells and assessed the basal level of the target protein by measuring GFP signal intensity using the Incucyte live cell imaging platform. This analysis revealed that 8 out of 10 variants exhibited significantly higher GFP levels, indicating reduced basal degradation compared to the control OsTIR1^F74G^ (Fig. 3a). Encouraged by these results, we further evaluated the functionality of these mutations by assessing GFP depletion following auxin treatment. This validation identified that 7 out of the 8 mutants (R20S, V63G, S210A, S210T, S217G, I533S, and D537G) maintained significant GFP depletion, thus confirming their functionality (Fig. 3b).

To better characterize these mutants, we knocked in OsTIR1^F74G^ with or without each of the top four mutants (V63A, S210A, S210T, V217A) into the AAVS1 safe harbor locus and generated clonal cell lines with homozygous knock-ins. These top mutants were selected based on having the highest basal GFP levels and significantly lower levels following auxin induction (Fig. 3c), indicating high on-target depletion efficiency. We also explored whether combinations of these mutants could further enhance acute depletion synergistically. We generated four different mutation combinations (S210A + V217A, S210T + V217A, V63A + S210A + V217A, and V63A + S210T + V217A), then knocked them into the *AAVS1* locus. Unexpectedly, none of these combinations improved relative depletion efficiency beyond that achieved with the single-point mutations (Fig. 3d).

Focusing on the S210A mutant, which exhibited minimal basal degradation and high depletion rates, we assessed whether this mutation in OsTIR1^F74G^ resulted in elevated basal target protein levels, efficient depletion, and, crucially, a higher recovery rate compared to the parental OsTIR1^F74G^ (Fig. 3e). Consistent with our screening strategy, flow cytometry analysis showed that S210A cells had significantly higher GFP signal intensity than control cells, indicating reduced basal degradation. We tested various auxin (5-PH-IAA) concentrations (10 nM to 1 μM) and confirmed that treatment with 1 μM 5-PH-IAA for 24 hours achieved complete target protein degradation (Fig. 3e). To compare the degradation kinetics of the new mutation, we temporally assessed GFP degradation dynamics in live cells using the Incucyte platform. Importantly, S210A demonstrated faster degradation kinetics over several time points following 1 μM 5-PH-IAA treatment (Fig. 3f).

#### Testing the sufficiency of OsTIR ^S210A^ mutant alone as an efficient AID degron.

Structural analysis facilitated by AlphaFold reveals that the S210 amino acid and the previously characterized F74 amino acid are located opposite each other in the β-sheet turning point that forms a central cavity in the OsTIR1 protein (Fig. 3g). Given that all previously identified mutations were tested in the OsTIR1^F74G^ background, we sought to determine whether the S210A mutation alone could function as an efficient degron without the F74G mutation. We thus engineered a new construct incorporating the S210A mutation in the absence of F74G and compared its performance against both wild-type OsTIR1 and OsTIR1^F74G^.

Remarkably, the S210A mutant alone demonstrated a comparable improvement in basal degradation to OsTIR1^F74G^, with both variants significantly outperforming wild-type OsTIR1 (Fig. 3h). However, further analysis indicated that S210A alone did not surpass F74G in terms of degradation kinetics or the need for lower auxin concentrations (Figs. 3i and 3j). These results suggest that while the S210A mutation enhances degradation compared to wild-type OsTIR1, its efficacy as a degron is significantly improved when synergistically combined with the F74G mutation. Thus, the combination of F74G and S210A mutations achieves minimal basal degradation, maximal depletion with auxin treatment, and optimal target protein recovery post-ligand washout. We have designated this refined degron as AID 3.0 and next planned to further validate its effectiveness on endogenous genes.

### AID 3.0 minimizes basal degradation and enhances target protein recovery of endogenous proteins

To evaluate the overall efficacy of AID 3.0, we created clonal cell lines with homozygous knock-ins of miniAID degrons at the C-termini of endogenous genes. This was achieved through nucleofection with Cas9 protein, gene-specific synthetic sgRNA, and a miniAID HDR template containing gene-specific homology arms. We miniAID-tagged the endogenous *DNMT3B* and *RBBP4* genes in parental cells expressing AID 2.0 (OsTIR1^F74G^) and AID 3.0 (OsTIR1^F74G + S210A^), where the respective OsTIR1 variants were previously knocked into the *AAVS1* locus. We then compared the relative efficiency of the two degron systems in terms of basal degradation, depletion kinetics following auxin treatment, and target protein recovery post-ligand washout.

**Figure 4:**
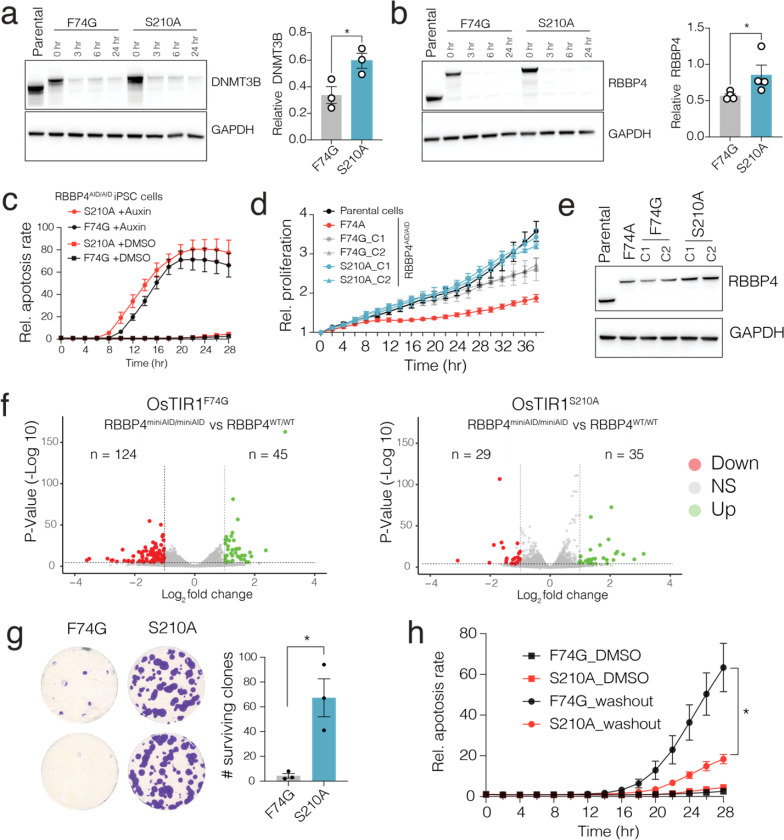
AID 3.0 enables minimal basal degradation, faster target depletion and dynamic recovery, overcoming limitations of current tools. **a-b)** The western blots show basal and induced depletion of endogenous degron tagged endogenous DNMT3B and RBBP4 proteins in iPSC cells expressing AID 2.0 (OsTIR1^F74G^) and AID 3.0 (OsTIR1^F74G + S210A^) after treatment 1 mM 5-Ph-IAA for indicated times. The bar plots show quantification of basal protein levels in both systems from three independent biological replicates. **c)** The line plot shows Incucyte live cell imaging data indicating relative apoptosis rates in degron-tagged-RBBP4 iPSCs expressing OsTIR1(F74G) or OsTIR1(S210A) after control and 1 mM 5-Ph-IAA treatment. **d)** The line plots show relative cell proliferation rates in degron-tagged-RBBP4 iPSC expressing OsTIR1 with F74G or F74G + S210A mutations and the wild-type parental cells without auxin treatment. **e)** Western blot show the basal RBBP4 protein levels in degron-tagged-RBBP4 iPSC expressing OsTIR1 with F74A, F74G or F74G + S210A mutations along with wild-type parental cells. **f)** The volcano plots show differential gene expression levels in degron-tagged RBBP4^miniAID/miniAID^ iPS cells expressing AID 2.0 (left panel) or AID 3.0 (right panel) relative to the parental RBBP4^WT/WT^ iPSC cells. **g)** Colony formation assay (quantified in bar plot)show the remaining surviving iPS cells after ligand washout (after 6 hr of 1 μM 5-Ph-IAA treatment) in degron-tagged-RBBP4 iPSCs expressing AID 2.0 or AID 3.0. **h)** The incucyte live cell imaging results show relative apoptosis rates in degron-tagged-RBBP4 iPSCs expressing AID 2.0 or AID 3.0 after auxin washout (after 3 hrs of 5-Ph-IAA treatment). Data and error bars in (a-d) and (g-h) indicate the means ± SD (*n* = 3–4 independent biological replicates). NS represents not significant, * represents *P* < 0.05, two-tailed *t*-test.

Significantly, a side-by-side comparison across multiple clonal cell lines demonstrated that AID 3.0 resulted in minimal basal degradation of both DNMT3B and RBBP4 proteins. In contrast, these proteins were significantly depleted in the uninduced state in AID 2.0 cells (Fig. 4a, 4b). Both degron systems efficiently depleted target proteins following 1 μM auxin treatment. Although the degradation kinetics and rates seemed comparable, AID 3.0 exhibited slightly improved degradation rates, particularly considering its ability to start with higher basal protein levels. This was evident when we studied the impact of acute protein deletion on cell viability and apoptosis. Knowing that RBBP4 is an essential gene (DepMAP database), we used the Incucyte live cell imaging platform to measure the kinetics at which cell will undergo cell death (measured by apoptotic Caspase cleavage) after inducing RBBP4 depletion. Notably, we observed that acute depletion of this protein led to significant apoptotic cell death in as little as 10 hours, indicating that RBBP4 is highly essential for cell survival (Fig. 4c). Importantly, cells with AID 3.0 had a more pronounced and faster apoptosis, indicating faster protein degradation dynamics in AID system (Fig. 4c).

#### AID 3.0 overcomes molecular and cellular defects due to basal degradation.

Observing this remarkable impact of RBBP4 depletion led us to quantify further whether the low basal degradation we observe in AID 2.0 will have any cellular impact. To this end, we measured long-term cell proliferation rates in WT parental cells as well as degron-tagged RBBP4 cells with AID 2.0 systems (OsTIR1^F74A^ and OsTIR1^F74G^) or AID 3.0 system (OsTIR1 ^F74G+S210A^). Notably, the proliferation rates of AID 3.0 expressing RBBP4^miniAID/miniAID^ cells were indistinguishable from the untagged parental cells (Fig. 4d). However, RBBP4^miniAID/miniAID^ cells with AID 2.0 systems showed significant proliferation defects (Fig. 4d). In line with these, we observed that the cells with AID 2.0 system had substantially higher basal degradation compared to AID 3.0 expressing cells, which maintained comparable RBBP4 protein levels with the parental untagged cells (Fig. 4e).

In line with proliferation defects in AID 2.0 system, we observed that the low basal degradation in the uninduced state results in ~ 170 differential expressed genes (p < 0.0001, Log2 fold change > 1) in these cells compared to parental cells (Fig. 4f, left panel). Notably, in line with the role of repressive role RBBP4 as part of number of repressive epigenetic complexes such as PRC2^30^, nucleosome remodeling and deacetylase (NuRD) complex^31^ and nucleosome remodeling factor (NURF) complex^32^, the majority of differentially expressed genes (124/45) were upregulated in degron tagged RBBP4 cells. Critically, under the same conditions, the number of differentially expressed genes was reduced from 169 genes to 64 genes in AID 3.0 expressing cells. Of the 124 upregulated genes, less than 25% of them were differentially expressed in AID 3.0 system, indicating that the improved degron system overcomes the significant number of molecular defects due to basal degradation observed in AID 2.0 system.

#### AID 3.0 enables faster target protein recovery.

In our screening strategy, we also aimed to generate OsTIR1 variants with faster target protein recovery (Fig. 2a). Given the highly lethal impact of acute RBBP4 protein depletion, we wanted to exploit this system to better quantify the impact and importance of a fast target protein recovery in washout experiments. To this end, we depleted RBBP4 in both AID 2.0 and 3.0 systems by treating with Auxin for 6 h and then removing the auxin by replenishing cells with fresh culture media. Critically, the acute RBBP4 depletion in AID 2.0 system results in near complete elimination of surviving iPSC clones after 48 h (Fig. 4g). In contrast, we observed robust recovery of growing iSPC clones that express the AID 3.0 system, confirming that our improved degron systems enable significantly faster recovery of the target protein after acute depletion. To further test this, we measured apoptotic cell death in cells after auxin washout. In line with Fig. 4g, we observed significantly less apoptosis in cells expressing AID 3.0 (Fig. 4h), further supporting that the re-engineered degron version enables faster target protein recovery, a vital property of an ideal degron system to perform rescue experiments even for highly essential genes.

## Discussion

Our study provides a comprehensive evaluation of four distinct degron technologies–dTAG, HaloPROTAC, auxin inducible OsTIR1 AID 2.0, and AtAFB2 tools for inducible protein degradation in human pluripotent cells. By comparing these technologies across multiple criteria—including basal degradation, inducible degradation kinetics, and recovery dynamics—we have identified critical strengths and limitations that guide the choice of degron systems for various experimental needs. Unlike previous studies that were either limited in their scope of tool comparison^12^ or used exogenously overexpressed degron-tagged cDNA rather than endogenous genes to compare the tools^18^, we established all the degron system in human pluripotent cells and used CRISPR to homozygous knock-in the needed degrons for the tool at the end of endogenous genes.

The auxin-inducible degron system, specifically AID 2.0, emerged as a highly effective tool for rapid and targeted protein depletion. However, several limitations were identified as critical weaknesses of this otherwise efficient degron technology. Specifically, we have observed a slower overall recovery rate compared to the AtAFB2 and HaloPROTAC approaches. It should be noted that the dTAG approach did not recover the target protein, making this approach the least effective for performing rescue-type biological experiments. Hence, we employed AID 2.0 (with OsTIR^F74G^) as potentially a more effective approach for large-scale null allele generation. However, upon larger scale validation of this tool, we noticed a substantial gene-specific basal degradation issue. This weakness, together with the slow recovery of target proteins with AID 2.0, prompted us to reengineer this system and evolve a superior degron technology.

Our base editing mediated saturating mutagenesis with a custom designed sgRNA library combined with rounds of functional selection strategy enabled discovery of several key gain-of-function mutations. Through such directed protein evolution and engineering, we demonstrate that the S210A mutation together with F74G creates a degron system, which we termed, AID 3.0 that significantly improves upon previous versions, particularly in minimizing basal degradation and enhancing target protein recovery dynamics. This advancement addresses one of the major limitations of earlier systems, where basal degradation could confound experimental results and impede accurate interpretation of protein function. Rapid target recovery enables the performing of rescue experiments in the same system, even for essential genes, where rapid recovery is critical for cell viability. AID 3.0’s combination of F74G and S210A mutations not only reduces leaky basal degradation but also ensures inducible rapid degradation and fast recovery of target proteins upon ligand removal. This substantiates AID 3.0 as a robust tool for studying dynamic biological processes and essential genes without the confounding effects of basal degradation.

Our study highlights the critical advancements achieved with AID 3.0 and its superiority over previous degron technologies in several key aspects. The enhanced performance of AID 3.0 represents a significant step forward in the field of targeted protein degradation, providing a more precise and controllable tool for functional genomics. It should be noted that all current degron systems work by fusing a “degron” peptide with endogenous proteins. This external peptide contains a substantial number of amino acid sequence that ranges from 68 aa for OsTIR systems to 107 aa for dTAG and 296 aa for the HaloTag system. It is likely that for certain genes, the addition of this stretch of external protein may alter the physiological function of the target protein. Therefore, future research should focus on minimizing the size of degrons by targeted engineering to obtain minimalist degrons that may minimally interfere with the protein function. Such improvements combined with the AID 3.0 system should expand the power of these tools and their applications to a broader range of cellular contexts and essential genes. With continued innovation and refinement, degron technologies, like AID 3.0, hold the promise of advancing our understanding of gene function and facilitating novel therapeutic approaches.

## Methods

### Plasmids construction:

Base editor plasmids pCMV_AncBE4max (Addgene, 112094) and pCMV_ABEmax (Addgene, 112095) were obtained from Addgene. The AAVS1_CAG_OsTIR1(F74G) mAID_EGFP KI plasmid was generated by restriction cloning with pMK411 (OsTIR1(F74G) mAID-EGFP-Nluc) cassette (Addgene,140659) and pMK381 (AAVS1 CMV-OsTIR1F74G) (Addgene,140536) backbone plasmid digesting by NdeI and MfeI-HF (New England Biolabs, R0111S, R3589S), following by removing useless DNA sequencing between two loxP sites with Cre Recombinase (New England Biolabs, M0298S). OsTIR1 wildtype and variants KI plasmids containing R20S, V63A, V63G, S210A, S210T, S210P, V217A, V217G, I533S, D537G substitutions were generated with AAVS1_CAG_OsTIR1(F74G)_mAID_EGFP as template using Q5^®^ Site-Directed Mutagenesis Kit (New England Biolabs, E0554S) according to the manufacturer’s protocol with modification, Q5 Hot Start High-Fidelity 2X Master Mix was replaced with Platinum^™^ SuperFi II PCR Master Mix (Invitrogen, 12368010). And all the substitutions in plasmids were verified by Sanger sequencing after site mutagenesis. AAVS1_CAG_OsTIR1(F74G)_PuroR, AAVS1_CAG_OsTIR1(S210A)_PuroR KI plasmids for parental cell line generation was cloned with AAVS1_CAG_OsTIR1(F74G)_mAID_EGFP, AAVS1_CAG_OsTIR1(S210A)_mAID_EGFP as backbones, and PCR fragment of puromycin selection marker from AAV:ITR-U6-sgRNA(LacZ)-pCBh-Cre-WPRE-hGHpA-ITR (Addgene, 60228) using restriction enzymes BglII (New England Biolabs, R0144S, ) and MfeI-HF. pSH-EFIRES-P-AtAFB2-mCherry-weak NLS plasmid was obtained from Addgene (129717). The degron KI template plasmids, AAVS1_CAG_OsTIR1(F74A) Loxp_mCherry_Loxp AAVS1_ EF1a_OsTIR1(F74A) Loxp_BSD_Loxp were synthesized by GenScript (NJ, USA).

### sgRNA preparation:

Saturating sgRNA sequences of OsTIR1 was identified using CHOPCHOP (https://chopchop.cbu.uib.no). Pooled oligo library was purchased from Twist Bioscience (CA, USA), then PCR amplified and cloned to BsmBI-digested lentiGuide-Puro backbone (Addgene, 52963) by Gibson Assembly as described^33^. Chemically modified sgRNAs for degron KI into CTCF, RAD21, DNMT3A and RBBP4 were purchased from Synthego. All sgRNAs sequences are provided in supplemental information.

### Cell culture:

HEK293T cells were cultured in Dulbecco’s Modified Eagle Medium (DMEM, Thermo Fisher Scientific, 11965092) containing 10% Fetal Bovine Serum (Fisher scientific, SH3091003) and 1% Penicillin–streptomycin (Life Technologies,15140–122). The KOLF2.2J iPS cell line was purchased from The Jackson Laboratory. The KOLF2.2J cells were maintained in Synthemax (Corning, 07–201-611)-coated or Matrigel (Fisher Scientific, 08–774-552)-coated in complete StemFlex media (Thermo Fisher Scientific, A3349401) without the addition of antibiotics according to JAX standard protocol with modification (supplemental materials). IPSCs were passaged using ReLeSR (Stem Cell Technologies, 05872) or dissociated with ACCUTASE (Stem Cell Technologies, 07920) Cells were cultured and incubated at 37 °C in a humidified atmosphere of 5% CO2 and 95% air. Cell lines were tested negative for mycoplasma using Lonza Walkersville MycoAlert PLUS Mycoplasma Detection Kit (Fisher Scientific, NC0447825).

### Lentivirus production and transduction:

HEK293T cells were seeded at 2.5 million cells in 10-cm dish until that reached to 50% confluence before packaging lentivirus. For packaging, 4 μg of Pooled sgRNA library, 2 μg psPAX2 (Addgene, 12260) and 1 μg of pmD2.G (Addgene,12259) were transfected using 21 μL polyethylenimine (PEI, 1mg/mL, Polysciences, 23966) in 600 μL OptiMEM (Thermo Fisher scientific, 51200038). Medium was replaced after 24 h. Lentivirus supernatants were collected at the 48 h and 72 h after transfection, filtered out cellular debris using Millipore’s 0.45-μm Stericup filter unit and stored at −80 °C. Serial dilutions of a virus were used to determine the lentiviral titer through transduction with 8 μg/mL polybrene and selection in 2 μg/mL puromycin for four days.

### Base editing screening:

HEK293T cells were seeded to be 70% confluent in 6-well plate before transfection. For base editing screening, 2.5 μg of pMK411 (OsTIR1(F74G) mAID-EGFP-Nluc) plasmid was transfected using Lipofectamine^™^ 3000 Transfection Reagent (Invitrogen^™^, L3000008) according to the manufacturer’s protocol. After 24 h, medium was replaced, and followed by selection with 200 μg/mL hygromycin (Sigma-Aldrich, H3274–50MG) for 7 days. A million of HEK293T cells after selection were transfected with the lentivirus expressing pooled sgRNA library at 0.25 MOI following by puromycin selection according to above-mentioned conditions. Successfully transduced cells that had stable sgRNA integration were transfected with 1.25 μg pCMV_AncBE4max (Addgene, 112094) and 1.25 μg pCMV_ABEmax (Addgene, 112095) in each well of 6-well plates using Lipofectamine^™^ 3000. Cells were then collected after 7 d, a million of collected cells were stored in −20 °C for DNA extraction, the rest of cells were sorted and collected top 20% GFP population at least 2.5 × 10^5^ cells (1000 x coverage) by FACSMelody 3-Laser Sorter (BD Biosciences) to seed in a 6-well plate. Once reaching to 80% confluence, cells were treated with 1 μM 5-Ph-IAA (MedChemExpress, HY-134653) for 1 h before sorting and collecting bottom 20% NO-GFP population. Sorted cells were brought back to recover for 3 days. Then washout cells were sorted and collected top 20% GFP recovery population to culture until getting enough for DNA extraction and another flow cytometry analysis. Data were subsequently analyzed using FlowJo version 10.

### DNA extraction, PCR and NGS sequencing:

For base editing screening, genomic DNA was extracted using the Purelink genomic DNA mini kit (Thermo Fisher, K182002) according to the manufacturer’s instructions. To avoid saturation of PCR amplification, qPCR was performed as following conditions: 98°C for 30 s, 40 cycles at 98 °C for 10 s, 63 °C for 30 s and 72 °C for 1 min, to determine the number of cycles. Each 50 μl PCR reaction for OsTIR1 and sgRNA sequence amplification was was performed with 1 μg of gDNA, 0.5 μM of forward 0.5 μM of reverse primer and 25 μl of NEBNext^®^ High-Fidelity 2X PCR Master Mix (New England BioLabs, M0541L) with the following cycling conditions: 98 °C for 30 s, 28 cycles of (98 °C for 10 s, 63 °C for 30 s, 72 °C for 1 min), followed by 72 °C for 5 min. All primers sequences are provided in the supplemental information. The PCR product was purified using QIAquick PCR Purification Kit (QIAGEN, 28106). Amplicon Sequencing Library of PCR products of OsTIR1 and sgRNA sequence was prepared by NUSeq Core Facility of northwestern university and sequenced using Illumina MiSeq (2 × 250 bp).

For identification of homozygous clones of CRISPR/Cas9 KI in IPSC, gDNA was extracted by adding 20 uL QuickExtract DNA Extraction Solution (Fisher Scientific, NC0302740) into Eppendorf tube contain clone cells. The samples were mixed by vortexing for 15 seconds, then transfered to a heat block at 65°C and incubated for 6 minutes. The samples were mixed again by vortexing for 15 seconds before being transferred to a heat block at 98°C for 2 minutes. Next, 20-μl PCR per reactions were performed with 0.5 μM of each forward and reverse primer, 2 μl of genomic DNA extract and 10 μl of Platinum^™^ SuperFiII PCR Master Mix (Invitrogen, 12368010). PCR reactions were carried out as follows: 98 °C for 30 s and then 35 cycles of 98 °C for 10 s, 60 °C for 10 s and 72 °C for 30 s per 1 kb, followed by a final 72 °C extension for 5 min. PCR products were evaluated analytically by electrophoresis in a 1 % agarose gel.

### Transfection and RNP nucleofection:

All OsTIR1 variants plasmids were first transfected to HEK293T cells at 1X10^4^ in 96-well plates using Lipofectamine 3000. The following day, we replaced medium and added with 1 μM 5-Ph-IAA to check GFP signal intensity change using Incucyte live cell imaging or treated for 24 h using flow cytometry. For KI of OsTIR1 variants into AAVS1 locus of HEK293T cells, a million of cells were nucleofected with 20 μg HiFi SpCas9 protein (IDT, 1081061), 8 μg chemical modified sgRNA (Synthgo) and 4 μg plasmid template using SF Cell Line 4D-Nucleofector^™^ X Kit L (Lonza, V4XC-2024) and the Lonza Amaxa 4D nucleofector (program DS150). For KOLF2.2J IPS cell line, a million of KOLF2.2J cells were nucleofected with 20 μg IDT HiFi Cas9 protein, 16 μg Synthetic sgRNA,120 μM Alt-R Cas9 Electroporation Enhancer (IDT, 10007805) and 10–15 μg plasmid template using P3 Primary Cell 4D-Nucleofector^®^ X Kit L (Lonza, V4XP-3024) and program CA137. Cas9 ribonucleoprotein complexes were assembled in vitro for 30 min at room temperature prior to nucleofection. Plasmid template was concentrated by ethanol participation and dissolved in P3 buffer. The nucleofected cells were seeded onto one well of a Synthemax-coated or Matrigel-coated 6-well plate in StemFlex media containing 1X ClonR2 (STEMCELL Technologies, 100–0691) and 1 uM Alt-R HDR Enhancer (IDT, 10007921) and cultured at 32°C (cold shock) for three days, then moved back to 37°C. After 24 h of nucleofection, the media was changed to remove HDR Enhancer and ClonR2. Cells were allowed to recover and grow for 5–7 d before puromycin selection or sorting by FACSMelody 3-Laser Sorter.

### Endogenous degron knock-in and single cell iPSCs clone generation:

IPSCs after puromycin selection were dissociated with Accutase and 2 X10^3^ were seeded to 10-cm Synthemax-coated dish in StemFlex media containing 1X ClonR2. For sorted cells, approximately 5 X10^3^ were seeded to 10-cm dish with StemFlex and 1X ClonR2, considering cells death during sorting. IPSCs clones were maintained to grow for 8–10 days. To avoid dead cells affecting PCR-genotyping, clones were washed 10 mL room temperature DPBS (Fisher Scientific, 14–190-144) twice and replaced with fresh media before picking up. Then each clone was manually split into two pieces using pulled Pasteur pipette (Fisher Scientific,1367820C). Half clone was seeded into Matrigel-coated 24-well plate in StemFlex media with 1X ClonR2, the remaining half of the clone was collected into Eppendorf tube with 200 μL DPBS, and centrifuged at 500g for 5 min, then media and DPBS were gently aspirated. Cells were stored in −20 °C for DNA Extraction.

### Incucyte live cell imaging:

Incucyte Live cell imaging system (Sartorius) was used for tracking cell proliferation and cell apoptosis. IPSCs were dissociated with Accutase and seeded to Matrigel-coated 96-well plate at 2X10^3^ in StemFlex media with 1X ClonR2. Next day, media was changed to remove ClonR2. At 48 h after seeding, cells were treated with 1 μM 5-Ph-IAA. For cell apoptosis, 0.5 μM NucView^®^ 530 Caspase-3 apoptosis dye (Biotium, 10406-T) was used and captured using the red channel. For washout experiments, cells were first treated with 1 μM 5-Ph-IAA for 3 h, then media with 5-Ph-IAA was removed and cells were washed once with DPBS before adding fresh media containing apoptosis dye. The system screened cells in the plate and took a photo using 10X objective every 2 h in different image channels (Phase, Green or Red). Proliferation data were analyzed using the Incucyte analysis tool and p-values were calculated using the Incucyte raw data. Relative proliferation was normalized to the starting time.

### Crystal violet assay for Auxin washout experiment:

Approximately 2 × 10^5^ IPSCs were seeded to each well of Matrigel-coated 6-well plate in StemFlex media with 1X ClonR2. After 24 h, media was replaced with fresh StemFlex media. After 48 h of seeding, IPSCs were treated with 1 μM 5-Ph-IAA for 6 h, then washed out residue media containing 5-Ph-IAA using DPBS and replaced with fresh StemFlex media. IPSCs after washing out were cultured for 10 days before performing crystal violet assay. Crystal violet assay was performed as previous study^34^. Briefly, cells were washed twice with distilled water. Then cells were fixed and stained by adding 0.5% crystal violet solution which was made by dissolving 0.5 g crystal violet powder in 100 mL 20 % methanol. After 20 min of incubation at room temperature on a bench rocker, fixed and stained cells were washed 3 times with distilled water, air-dried overnight and imaged by the scanner. The viability of IPSCs after washout was determined by colony counting using ImageJ.

### Western Blotting:

Cells were washed once with cold DPBS, then immediately scraped off the plate and transferred to a microcentrifuge tube, centrifuged at 500g in 4°C for 5 min, then washed again using cold DPBS. Cells were mixed with 1X RIPA buffer (Boston BioProducts, BP-115) containing 1X Protease Inhibitor Cocktail (Thermo Scientific, 78430) by vertexing every 10 min, and lysed on ice for total 40 minutes. Or cells were lysed by sonication for 30 s. Protein concentrations were determined using the BCA assay (23225, Thermo Fisher). Protein was mixed with 4X Laemmli reducing sample Buffer (Boston BioProducts, BP-110R) and boiled at 95 °C for 10 min. Equal amounts of boiled lysates were loaded onto either a 4–12%, NuPAGE^™^ Bis-Tris Mini Protein Gels (Thermo Fisher, NP0335BOX) or 7.5% Tris-Glycine Gels. Proteins were transferred to iBlot Nitrocellulose Transfer Stack (Fisher Scientific, IB301002) membrane using iBlot dry transfer system with Program 3 for 7 min or PVDF membrane (Bio-Rad, 1620174) using wet transfer in 4°C for overnight. Membranes were blocked using 5% milk dissolved in TBS-T (20 mM Tris, 150 mM NaCl, 0.1% Tween 20; pH 7.6) for 1 h at room temperature. Membranes were incubated with primary antibodies (1:1000 dilution) for overnight at 4 °C. Primary antibodies including RAD21 (Cell Signaling, 4321S), CTCF (Cell Signaling, 2899S), DNMT3B (Cell Signaling, 67259), RBAP46/RBAP48 (Cell Signaling, 9067), GAPDH (Cell Signaling, 2118) were used in this study. The next day, membranes were washed three times for 5 min with TBS-T, then incubated with secondary antibodies (1:10,000) (Anti-Rabbit IgG (H + L) (Promega, W4011) or Anti-rabbit IgG, HRP-linked Antibody (Cell Signaling, 7074) diluted in blocking buffer for 1 h at RT. After the incubation, membranes were washed three times for 10 min with 10 ml of TBS-T. Membranes were covered with ECL Prime Western Blotting Detection Reagent (Cytiva, RPN2232) or SuperSignal^™^ ELISA Femto Substrate (Thermo Fisher, 37074) and visualized using the iBright imaging system. Western blots images were analyzed using iBright Imaging Systems and Photoshop to quantify the protein.

### Structure analysis via AlphaFold2:

OsTIR1 protein structures were predicted using Alphafold2.

### Mutational analysis of OsTIR1 and sgRNA enrichment analysis:

The quality control of the FASTQ files was performed using FastQC (v0.12.0), followed by trimming low-quality reads by Trimmomatic (v0.39). CB2 (CRISPR Beta Binomial) was used to identify and quantify sgRNAs. Enrichment and depletion were determined based on z-scores. The OsTIR1 sequencing reads were aligned to the reference OsTIR1(F74G) genome using Bowtie2. The SAM files were sorted to BAM files using SAMtools (v1.21). Then, the BAM files were converted to the BigWig files using DeepTools (v3.5.1). The BigWig files were visualized in the Integrative Genomics Viewer (IGV, v2.11.4) to identify the frequencies of mutants.

### Bulk RNA-Seq and the data analysis:

Total RNA was isolated using the Quick RNA miniprep kit (Zymo research, R1057) with DNase I treatment by following the manufacturer’s instructions. The integrity of total RNA was assessed through 1% TAE agarose gel electrophoresis. mRNA was isolated using NEBNext Poly(A) mRNA Magnetic Isolation Module (New England Biolabs, E7490L). RNA-Seq libraries were prepared using the NEBNext^®^ Ultra^™^ II Directional RNA Library Prep Kit for Illumina^®^ (New England Biolabs, E7760L) and NEBNext Multiplex Oligos (NEB, E7500L) with index primers according to the manufacturer’s instructions. The concentration of all indexed PCR products was measured by the qubit before pooling equal amount to the library. And bioanalyzer was used to determine the library quality. RNA-seq library sequencing was performed by NUSeq Core Facility using Aviti PE75 Sequencing.

The quality of sequencing FastQ files were checked using FastQC. The reads were aligned to the GENCODE human genome V46 ( GRCh38.p14) by the STAR aligner (v2.7.9a) with default parameters. Differential expression analysis was performed using R package DESeq2 (V1.44.0) Benjamini-Hochberg correction for multiple testing. The false discovery rate (FDR) was controlled at *P* < 0.0001. The volcano plots were generated using R package EnhancedVolcano (v1.22.0).

### Embryoid bodies (EB) formation, maintenance and dissociation:

EBs were formatted and maintained with the STEMCELL Aggrewell400 protocol with modification. The Aggrewell 400 24-well plate (Fisher Scientific, 34450) was coated with anti-adherence rinsing solution (STEMCELL Technologies, 07010). Then the CAG-OsTIR1, EF1a-OsTIR1 and EF1α_ATAFB2 parental cell lines were dissociated with Accutase, mixed equally and seeded into to the coated Aggrewell 400 24-well plate at 1.2 × 10^6^ per well (1000 cells per microwell) in Aggrewell EB Formation Medium (STEMCELL Technologies, 05893). Half of the media with fresh Aggrewell EB Formation Medium after 24 h. At 48 h after seeding, EBs were harvested and moved to ultra-low attachment six-well plate in Essential 6^™^ (E6) Medium (Thermo Scientific, A1516401). EBs were maintained in culture for 19 days by replacing with fresh E6 media every other day. EBs were collected and dissociated at day 21. The collected EBS were washed with DPBS once, then treated with Accutase and incubated at 37 °C. After 10 min, EBs in Accutase were dissociated by pipetting with P1000 tip for 30 s every 5 min until EBs were completely dissociated. E6 media was added to terminate the dissociation, and cells were filtered through a 40 μM strainer (Fisher Scientific, 08–771-1) before counting.

### Single-cell RNA sequencing:

EBs dissociated cells were used for scRNA-seq library preparation with a targeted collection of 1 × 10^4^ cells. Single-cell 3’ RNA-seq libraries were generated with 10x Genomics Chromium Next GEM Single Cell 3’ Reagent Kit v.3 following the manufacturer’s guidelines. The libraries were sequenced on platform NovaSeq S2 PE50 sequencing by NUSeq Core Facility.

### Single-cell data analysis:

Single-cell RNA-seq FASTQ files were aligned to a custom version of the human reference genome GRCh38 using Cellranger Version 8.0.1 and default parameters. The reference genome was created using the Cellranger mkref command and had 3 transcripts appended to it representing unique sequences present in the three constructs that needed to be detected (CAG-OsTIR1, EF1a-OsTIR1, and EF1a-AtAFB2) (Supplement File). The resulting count matrices were subsequently analyzed in R using Seurat Version 5.0.3. Following QC, the cells were labeled based on which of the three constructs they were expressing. Any cells that were erroneously shown to be expressing more than one (likely doublets) were removed.

### Statistics and reproducibility:

Statistical analyses were performed using GraphPad Prism version (10.2.3). Descriptive statistics are reflected as means ± standard deviations (SDs). Sample sizes (*n*) and analyses for all experiments are defined in the appropriate main or supplementary figure legends.

## Supplementary Material

Supplement 1

## Data Availability

All genomic data produced in this manuscript will be deposited in publicly accessible repositories before publication.
