# Supplementary material for: Comparative analysis and directed protein evolution yield an improved degron technology with minimal basal degradation, rapid inducible depletion, and faster recovery of target proteins
Source: Res Sq. 2024 Nov 15:rs.3.rs-5348956. Preprint. [Version 1] doi: 10.21203/rs.3.rs-5348956/v1 (PMC11601833; doi:10.21203/rs.3.rs-5348956/v1)
Supplement: Supplement 1 [file NIHPPRS5348956V1-supplement-1.pdf]

Supplementary Files

This is a list of supplementary files associated with this preprint. Click to download.

- SupplementaryfiguresFinal.docx
- supplementalinformation.xlsx
- epcma.pdf
